# Similar severity of influenza primary and re-infections in pre-school children requiring outpatient treatment due to febrile acute respiratory illness: prospective, multicentre surveillance study (2013–2015)

**DOI:** 10.1186/s12879-021-06988-7

**Published:** 2022-01-04

**Authors:** Andrea Streng, Christiane Prifert, Benedikt Weissbrich, Andreas Sauerbrei, Andi Krumbholz, Ruprecht Schmidt-Ott, Johannes G. Liese

**Affiliations:** 1 Department of Pediatrics, University Hospital of Würzburg, Josef-Schneider-Str. 2, 97080 Würzburg, Germany; 2 Institute for Virology and Immunobiology, University of Würzburg, Würzburg, Germany; 3 Section Experimental Virology, Institute of Medical Microbiology, Jena University Hospital, Jena, Germany; 4 Institute for Infection Medicine, Christian Albrecht University of Kiel and University Medical Centre of Schleswig Holstein, Kiel, Germany; 5 GSK, Wavre, Belgium

**Keywords:** Influenza, Children, Disease severity, IgG, Immunology

## Abstract

**Background:**

Influenza virus infections in immunologically naïve children (primary infection) may be more severe than in children with re-infections who are already immunologically primed. We compared frequency and severity of influenza virus primary and re-infections in pre-school children requiring outpatient treatment.

**Methods:**

Influenza-unvaccinated children 1–5 years of age presenting at pediatric practices with febrile acute respiratory infection < 48 h after symptom onset were enrolled in a prospective, cross-sectional, multicenter surveillance study (2013–2015). Influenza types/subtypes were PCR-confirmed from oropharyngeal swabs. Influenza type/subtype-specific IgG antibodies serving as surrogate markers for immunological priming were determined using ELISA/hemagglutination inhibition assays. The acute influenza disease was defined as primary infection/re-infection by the absence/presence of influenza type-specific immunoglobulin G (IgG) and, in a second approach, by the absence/presence of subtype-specific IgG. Socio-demographic and clinical data were also recorded.

**Results:**

Of 217 influenza infections, 178 were due to influenza A (87 [49%] primary infections, 91 [51%] re-infections) and 39 were due to influenza B (38 [97%] primary infections, one [3%] re-infection). Children with “influenza A primary infections” showed fever with respiratory symptoms for a shorter period than children with “influenza A re-infections” (median 3 vs. 4 days; age-adjusted p = 0.03); other disease characteristics were similar. If primary infections and re-infections were defined based on influenza A subtypes, 122 (87%) primary infections (78 “A(H3N2) primary infections”, 44 “A(H1N1)pdm09 primary infections”) and 18 (13%) re-infections could be classified (14 “A(H3N2) re-infections” and 4 “A(H1N1)pdm09 re-infections”). Per subtype, primary infections and re-infections were of similar disease severity. Children with re-infections defined on the subtype level usually had non-protective IgG titers against the subtype of their acute infection (16 of 18; 89%). Some patients infected by one of the influenza A subtypes showed protective IgG titers (≥ 1:40) against the other influenza A subtype (32/140; 23%).

**Conclusions:**

Pre-school children with acute influenza A primary infections and re-infections presented with similar frequency in pediatric practices. Contrary to expectation, severity of acute “influenza A primary infections” and “influenza A re-infections” were similar. Most “influenza A re-infections” defined on the type level turned out to be primary infections when defined based on the subtype. On the subtype level, re-infections were rare and of similar disease severity as primary infections of the same subtype. Subtype level re-infections were usually associated with low IgG levels for the specific subtype of the acute infection, suggesting only short-time humoral immunity induced by previous infection by this subtype. Overall, the results indicated recurring influenza virus infections in this age group and no or only limited heterosubtypic antibody-mediated cross-protection.

**Supplementary Information:**

The online version contains supplementary material available at 10.1186/s12879-021-06988-7.

## Background

Influenza in humans is mainly caused by infections with influenza virus type A (“influenza A”), with two currently circulating subtypes A(H3N2) and A(H1N1)pdm09, and by influenza virus type B (“influenza B”), with the two genetically distinct lineages, influenza B/Victoria and B/Yamagata, circulating in Europe [1, 2]. During childhood, influenza usually presents as a mild, febrile, acute respiratory infection (ARI) treated in outpatient practices, with low rates of complications such as pneumonia, otitis media, encephalitis, or neuromuscular disease [3–5]. In outpatient settings, clinical characteristics and disease severity of infections with different influenza virus types and subtypes appear similar in children of similar age [2, 6–8].

However, little is known of the impact of natural immunological priming on clinical disease severity in children, and the natural acquisition and persistence of type and subtype-specific antibodies directed against influenza virus serving as surrogate markers for priming. Passively acquired maternal immunoglobulin G (IgG) antibodies against influenza may protect newborns and infants against infection with influenza virus, but they usually wane within the first months of life [9, 10]. During this immunologically naïve period, the first-ever (primary) natural influenza virus infection in children may therefore result in a particularly severe course of disease [11]. Influenza IgG antibodies likely appear after a period of 5 to 10 days after infection [12, 13] and are supposed to be reliable surrogate markers for acquired immune protection, which may rely primarily on cell-mediated immunity [11, 14]. If IgG antibodies are present in sufficiently high titers, they are expected to protect against re-infections and influenza disease by the same influenza virus subtype strain later in life [11, 15, 16]. However, IgG antibodies against a specific influenza virus subtype strain may also show cross-reactivity against related other strains of the same subtype (homosubtypic cross-reaction) [17] or even against other subtypes (heterosubtypic cross-reaction) to a varying extent, depending on the degree of genetic divergence of the strains, patient age and the sequence of subtype infections [14, 15, 17]. Interestingly, for young children a recent study on influenza virus A(H1N1)pdm09 infections demonstrated that natural infection induced only a very narrow, homosubtypic influenza virus antibody response, putting into question the extent of antibody-mediated cross-protection to later re-infection [14].

Seroprevalence studies in unvaccinated children based on the absence or presence of IgG antibodies directed against influenza A and influenza B or an influenza subtype/lineage reflect the likelihood of primary infection with each influenza (sub)type/lineage at a certain age. In Germany, seroprevalence data indicated contact with influenza A early in life, with more than 75% of children aged 3–6 years shown to be seropositive for this type, while children remained seronegative much longer for influenza B due to its lower circulation in the population [18]. A seroprevalence study conducted in Germany during the first months following the 2009/2010 pandemic caused by the newly emerged A(H1N1)pdm09 showed an infection rate of 25% in the population of children < 5 years of age who were immunologically naïve for this subtype [19].

During the years following the influenza pandemic of 2009/2010, renewed interest arose regarding the key role of influenza virus primary infection on influenza virus immunity later in life. This was based on the observation that elderly people were less affected by this pandemic than other age groups, presumably due to contact with a related A(H1N1) strain in their early childhood resulting in homosubtypic cross-reactivity. Roughly 60 years ago, it had already been hypothesized that influenza primary infection in childhood had a lasting effect on the immune system (‘antigenic imprinting’), resulting in a life-long higher strength of protection against the imprinting subtype [15]. Several recent immunological, surveillance and modeling studies support this hypothesis and discuss its potential implications for future influenza epidemiology, surveillance and vaccination strategies [14, 16, 20–23].

Despite this expected high immunological importance of influenza virus primary infections, the clinical disease characteristics and severity of natural influenza virus primary infections compared to re-infections in young children are largely unknown, and detailed information on the antibody status in young children with an acute natural influenza infection are lacking.

In the present study, we investigated pre-school children requiring outpatient treatment due to acute febrile influenza virus infection. We first determined the frequency of influenza virus primary infections and re-infections on the type and on the subtype/lineage level, and evaluated influenza virus IgG antibody titers on the type and on the subtype/lineage level to assess the potential for cross-protection among subtypes/lineages. For influenza types and subtypes with sufficient numbers of primary and re-infections detected, we compared primary and re-infections regarding their clinical characteristics, impact on the family and healthcare utilization.

## Methods

### Study setting and conduct

Details of the study setting, the procedures and the clinical data collection were already published [8]. In brief, we conducted a prospective, cross-sectional surveillance study in 33 outpatient pediatric practices in Southern Germany during three influenza seasons (2012/2013–2014/2015). Children 1–5 years of age presenting at the practice with febrile acute respiratory infection (body temperature ≥ 38.0 °C plus rhinitis and/or cough) were enrolled and an oropharyngeal swab and a blood sample were taken from each child. Excluded were children below 1 year of age (to avoid misclassification through potential presence of maternal influenza IgG antibodies), children vaccinated against influenza at any time (potential presence of vaccine-induced influenza IgG antibodies), and children presenting ≥ 48 h after onset of acute respiratory symptoms (potential presence of influenza IgG antibodies developed early during the acute influenza virus infection). Additionally, children who had already participated in the study and presented later again at the pediatric practice with a new episode of acute respiratory infection were not included a second time. The physician documented sociodemographic data, clinical assessments and healthcare utilization at the initial visit (Day 0) and during follow-up telephone calls at Day 7 and Day 14 using a questionnaire. Follow-up information was obtained from a patient diary filled in by the parents until the end of the acute respiratory symptoms. In addition, the physician collected the patient’s history of previous acute and chronic respiratory diagnoses from the pediatric practice files.

The main outcome measure was disease severity, defined as duration of disease with flu-like symptoms (days with body temperature ≥ 38.0 °C plus either cough or rhinitis) [24]. The course of disease was evaluated using a parental, 18-item severity score (“Canadian Acute Respiratory Illness and Flu Scale”, CARIFS) [25] on Day 0, Day 3 and Day 6, with illness severity expressed by a 54-points sum score (0 = best, 54 = worst possible health).

### RT-PCR to determine the influenza virus type/subtype of the acute infection

Oropharyngeal swabs were placed in a viral transport medium (Mast Diagnostica GmbH, Germany) and tested at the University of Würzburg, Institute for Virology and Immunobiology for influenza A, influenza B and other common viral respiratory pathogens, using multiplex PCR (‘FTD^®^ Respiratory Pathogens 21’, Fast Track Diagnostics, Luxembourg) [8]. The tests were performed according to the instructions of the manufacturer using ‘AgPath-IDTM One-step RT-PCR reagent’ (Life Technologies, Darmstadt, Germany) as PCR master mix on 7500 real-time PCR thermocyclers (Life Technologies). For samples positive for influenza virus A RNA, primers and probes specific for A(H1N1)pdm09 were included in the test kit, and A(H1N1)pdm09-negative samples were re-tested by influenza virus A H3-specific PCR (for details, see Additional file 1: Additional methods). Cycle threshold values < 25/25–35/> 35 indicated high/medium/low viral load.

### ELISAs and hemagglutination inhibition assays to determine influenza virus IgG antibody prevalence from previous influenza virus infections

Blood serum samples were tested at the University Hospital of Jena, Section Experimental Virology (formerly Institute of Virology and Antiviral Therapy) using influenza virus A-specific and influenza virus B-specific ELISAs to determine IgG antibody prevalence. Sera were tested in parallel using kits of influenza virus A IgG ELISA (IBL International, Hamburg, Germany) and influenza virus B IgG ELISA (Euroimmun, Lübeck, Germany). Both ELISAs were carried out manually and used for qualitative and semi-quantitative antibody testing. All samples were tested twice.

The influenza virus A IgG ELISA used whole, inactivated influenza virus A Sydney/5/97 (H3N2) and Beijing/262/95 (H1N1) and the influenza virus B IgG ELISA used whole, inactivated influenza virus B Hongkong/5/72 as antigens in pre-coated micro-titration strips. The antigens contained high amounts of conserved influenza virus type-specific nucleo- and matrix proteins. Testing of sera was carried out according to the manufacturers’ instructions for use. Results were assessed based on a standard curve calculated from three to four calibrators including positive and negative controls. Using influenza virus A IgG ELISA, samples were considered positive if antibody concentration was calculated as > 12 U/ml, a range of 8–12 U/ml was considered equivocal and < 8 U/ml was interpreted as negative. For influenza virus B IgG ELISA, samples were considered positive if antibody concentration was calculated as ≥ 22 RE/ml, a range of ≥ 16 to < 22 RE/ml was considered equivocal and < 16 RE/ml were interpreted as negative. The diagnostic sensitivity and specificity of the IBL influenza virus A IgG ELISA was reported by the manufacturer to be > 95%, whereas no exact manufacturer information was available for the Euroimmun influenza virus B IgG ELISA. Therefore, both ELISAs were tested together with other commercially available type-specific influenza virus ELISAs in comparison to the hemagglutination inhibition assay [26] by including defined serum samples from children [27]**,** newborns and their mothers [9]. Since both the IBL Influenza virus A IgG ELISA and the Euroimmun influenza virus B IgG ELISA revealed sensitivities ≥ 97% and no cross-reactivities between influenza virus A and influenza virus B or to other viral pathogens [18], the ELISAs were selected as the most sensitive and specific. This approach had already proven successful in a large study to determine prevalence of influenza virus A and influenza virus B antibodies in German children [18].

In sera with positive influenza virus-A or influenza virus-B IgG ELISA results, the influenza virus A subtype-specific or influenza virus B lineage-specific antibody response was determined by the hemagglutination inhibition (HI) assay. The following influenza virus strains were used as antigens: A/Kiel/200001978/2013 (seasonal H3N2, A/Victoria/361/2011-like), A/Kiel/20002079/2013 (pandemic H1N1 2009, A/California/07/2009-like), B/Kiel/20003315/2013 (Yamagata lineage, B/Wisconsin/1/2010-like) and B/Jena/20001499/2011 (Victoria lineage, B/Brisbane/60/2008-like) were used for testing sera obtained in the seasons 2012/2013 and 2013/2014. A/Kiel/18044827/2015 (seasonal H3N2, A/Texas/50/2012-like), A/Kiel/18034557/2015 (pandemic H1N1 2009, A/California/07/2009-like) and B/Kiel/18052717/2015 (Yamagata lineage, B/Massachusetts/2/2012-like) were included as additional viral strains for sera from the 2014/2015 season.

All strains were isolated and passaged in Madin-Darby canine kidney (MDCK) cells. Cells were propagated in Dulbecco’s modified Minimum Essential Medium (DMEM) supplemented with 10% (v/v) fetal bovine serum (FBS), 100 U/ml penicillin, 100 μg/ml streptomycin and 2 mM l-glutamine. The FBS was removed for influenza virus isolation, and 3 µg/mL trypsin as well as 25 mM MgCl_2_ were added [28]. The HI assay was performed as an in-house modification of the standard WHO protocol described previously [26, 29]. Serum samples were pre-treated for 14–18 h at 37 °C with neuraminidase (Sigma-Aldrich N-3001, Munich, Germany) diluted 1:40 with 0.9% (w/v) sodium chloride. It was followed by enzyme inactivation by incubation with 1.5% (w/v) sodium citrate for 30 min at 56 °C and the hemadsorbtion of sera with chicken erythrocytes for 1 h at 4–8 °C. Then, the sera were serially diluted two-fold, beginning with 1:20, in microtiter plates, and 25 μl of serum dilution were each incubated with standardized antigen in a concentration of 8 hemagglutinating units at 20–25 °C for 45 min. This was followed by a further incubation with a standardized solution of 0.5% chicken erythrocytes at 20–25 °C for 30 min. Sera and red blood cell controls were included in each experiment. Finally, the HI titers were calculated as the highest dilution of serum that inhibited virus-induced hemagglutination. All HI assays were carried out twice on each virus, and geometrical mean of the respective HI titer was calculated for each serum. A titer ≥ 1:40 was considered as usually protective against the respective influenza virus subtype/lineage [19].

### Definition of the acute influenza disease as either primary infection or re-infection

Following infection with influenza virus, the first symptoms of disease appear after 1 to 3 days and influenza virus IgG antibodies appear usually after a period of 5 to 10 days [12, 13]. Hence, the influenza infection leading to the pediatric practice visit was classified as a primary infection if a blood sample collected < 48 h after onset of acute respiratory symptoms contained no specific influenza virus IgG. Accordingly, presence of specific influenza virus IgG indicated previous infection(s) and, thus, the acute influenza disease was classified as a re-infection.

#### Definition of primary infections and re-infections on the influenza type level

As a first approach, primary infections and re-infections were defined based on the influenza type, i.e. for acute influenza virus A infection by the absence (primary infection) or presence (re-infection) of (any) influenza virus A IgG, and for (any) influenza virus B infection by the absence or presence of (any) influenza virus B IgG, respectively. For example, if a child attended the pediatric practice due to influenza A infection, this acute infection was classified as “influenza A primary infection” if the blood sample drawn at the practice visit revealed no influenza A-specific IgG. If there was any influenza A IgG, the acute disease was classified as an “influenza A re-infection”, regardless of the influenza A subtype.

#### Definition of primary infections and re-infections on the influenza subtype level

As a second approach, primary infections and re-infections were defined based on the level of the influenza virus subtype/lineage, i.e. for the subtype/lineage-specific acute infection by absence or presence of IgG antibodies only for this specific subtype/lineage. For example, if a child attended the pediatric practice due to an acute influenza A(H3N2) infection, this infection was classified as “A(H3N2) primary infection” if the blood sample drawn at the initial practice visit revealed no influenza A(H3N2) IgG (regardless of the presence or absence of A(H1N1)pdm09 IgG). If there was any influenza A(H3N2) IgG present, the disease was classified as an “A(H3N2) re-infection”.

Therefore, some acute infections classified on the influenza type level as “influenza A re-infections” during the first approach were then re-classified as “primary subtype infections” for the specific influenza A virus subtype in the second approach.

### Sample size calculation

The study aimed at recruiting 800 patients with febrile, acute respiratory infections. This was based on the following assumptions: (a) 20% of patients with acute respiratory infections are infected with influenza virus; (b) the ratio of influenza virus primary infections to re-infections in the investigated age group is 1:1; (c) the duration of disease with flu-like symptoms (main outcome criterion) for influenza primary infections compared to re-infections is increased by 2 days (7/5 days ± SD 3 days for primary infections/re-infections); (d) a drop-out rate of 35% due to invalid documentation or laboratory specimens. Given these assumptions, a sample size of 50 children with laboratory-confirmed influenza virus primary infections and of 50 with influenza virus re-infections were estimated to provide 90% power to reject a two-sided test at a significance level of 0.05.

### Statistical analysis

All data were transferred into IBM SPSS 21.0 for statistical analysis. Data were analysed descriptively (counts and percentages, or median with inter-quartile range, IQR). Exploratory comparisons among groups were assessed for significance (p < 0.05, two-sided) using Pearson’s Chi2-test or, where appropriate, Fisher’s Exact test for categorical data. Continuous data were compared using the Mann–Whitney U-test. Additionally, multivariable Poisson regression models were used to compare the disease severity (measured as days with flu-like illness as main outcome criterion) between children with influenza primary infections and re-infections, adjusting for pre-defined potential confounder variables (age, sex, underlying chronic condition, siblings, childcare attendance, influenza type/subtype, viral co-infection, influenza season). In a sensitivity analysis, zero-truncated Poisson regression models were fitted to account for the fact that the main outcome criterion was by definition strictly positive. The multivariable analyses were carried out in R for Windows 3.2.3.

## Results

### Patient enrollment and study inclusion

**Fig. 1:**
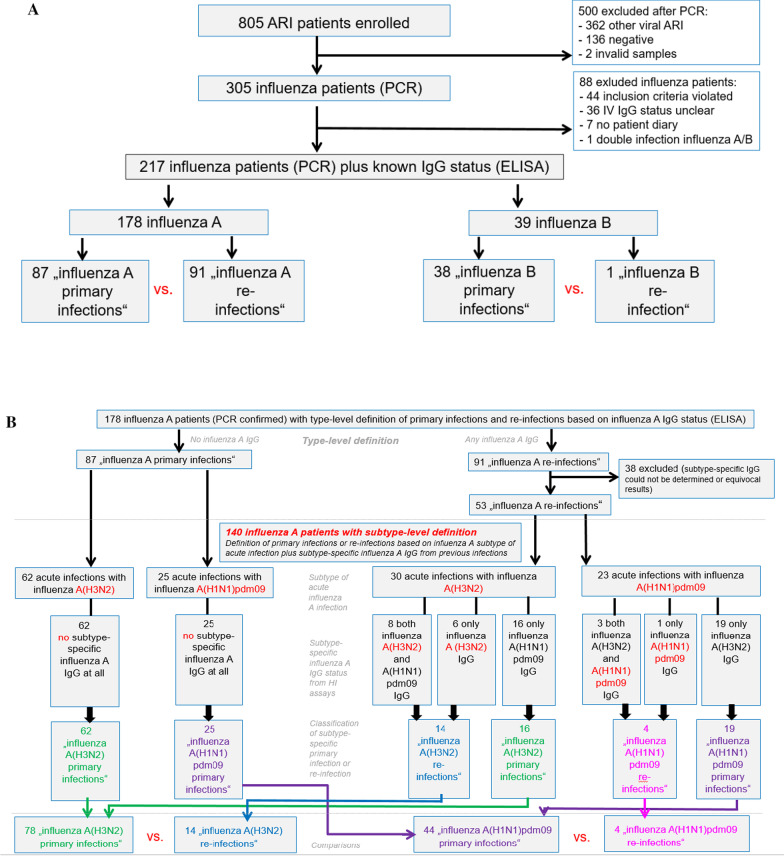
Study flow chart. Patients were recruited from outpatient pediatric practices in Bavaria (Germany), 2013–2015. Children aged 1–5 years with febrile acute respiratory infection were enrolled. Patients with PCR-confirmed influenza virus infection were included for the present analyses. Part **A** Classification of the acute influenza disease as either primary infection or re-infection was defined first on the influenza virus type-level using ELISA to determine influenza A IgG and influenza B IgG serostatus. Part **B** For patients with acute influenza A infection, in a second approach, primary infection or re-infection were defined on the influenza A virus subtype level using Hemagglutination inhibition (HI) assays to determine subtype-specific IgG serostatus. Note that “influenza A re-infections” defined on the type-level may represent primary infections for a specific influenza A subtype, and if so, are re-classified as either “A(H3N2) primary infection” or “A(H1N1)pdm09 primary infection” for analyses on the subtype level

Overall, 805 patients with febrile acute respiratory infection were enrolled, and acute influenza infection was PCR-confirmed for 305 (37.9%) children (Flow chart, see Fig. 1, Part A; for details see [8]). A total of 217 (71.1% of 305) influenza patients with known subtype plus ELISA-confirmed IgG status could be included in the present analyses. A total of 178 (82.0% of 217) children were acutely infected with influenza A (including 122 with A(H3N2) and 56 with A(H1N1)pdm09) and 39 (18.0%) with influenza B. Details of overall and (sub)type-specific socio-demographic and clinical characteristics of the 217 patients were published elsewhere [8].

### Serological status of patients with acute influenza A or influenza B infections, and the resultant classification of primary infections and re-infections on the type and on the subtype level

#### Serological status (IgG) of influenza A patients on the type level (ELISA)

On the type level, 87 (48.9%) of 178 included influenza A patients had an “influenza A primary infection” and 91 (51.1%) an “influenza A re-infection”, based on ELISA-confirmed absence or presence of influenza virus A IgG.

#### Serological status (IgG) of influenza A patients on the subtype level (HI assay)

For a subsample of 140 (78.7% of 178) patients with PCR-confirmed acute influenza A infection (92 with influenza A(H3N2), 48 with influenza A(H1N1)pdm09) subtype-specific influenza A IgG could be determined by the HI assays (Table 1; Fig. 1, Part B).

**Table 1:** Antibody status in children with acute subtype-specific influenza primary infections and re-infections, with primary and re-infections defined according to the absence or presence of subtype-specific IgG in HI assays

	Children treated in outpatient practices for acute influenza A virus infection, by subtype			
	Acute A(H3N2) infections		Acute A(H1N1)pdm09 infections	
	Primary infections	Re-infections	Primary infections	Re-infections
	N = 78	N = 14	N = 44	N = 4
Previous influenza infection indicated by IgG antibody status				
No influenza A IgG	62	–	25	–
A(H3N2) IgG	–	**14**	19	3
Titer		***13 with non-protective titer (*< *1:40), 1 with protective titer***^***a***^*** (1:160)***	*7 with non-protective titer, (*< *1:40), 12 with protective titer (1:40–1:450)*	*1 with non-protective titer (*< *1:40), 2 with protective titer (1:160–1:320)*
A(H1N1)pdm09 IgG	16	8	–	**4**
Titer	*2 with non-protective titer (*< *1:40), 14 with protective titer (1:40 to 1:320)*	*4 with non-protective titer (*< *1:40), 4 with protective titer (1:40–1:320)*		***3 with non-protective titer (*< *1:40), 1 with protective titer***^***a***^*** (1:80)***

Data from a subgroup of 140 influenza A outpatients aged 1–5 years, with known influenza virus subtype of the acute infection and subtype-specific influenza A IgG antibody determination. Influenza A IgG presence indicates a previous infection with a specific influenza A subtype. Bold letters in the columns “Re-infections” indicate patients with IgG antibodies against the subtype of the acute infection, thus determining the classification as subtype-specific “re-infection”. For these patients, information in bold italic letters describes the level of protection according to the IgG antibody titer. By definition, in acute subtype-specific influenza A primary infections, there are no IgG antibodies against the specific subtype causing the disease; however; these patients may possess IgG against the other influenza A subtype

For each IgG subtype, the number of patients with non-protective or normally protective titers were reported. Numbers in brackets refer to IgG antibody titers from hemagglutination inhibition assays (titer of ≥ 1:40 considered ‘protective’ against infection with the respective subtype)

Note that the IgG antibody status is presented only for influenza virus A. For those children in Table 1 with detected influenza virus A titers, the titers for lineage-specific influenza virus B IgG were additionally evaluated; of these patients, 40 had also IgG against at least one influenza virus-B lineage (details not shown)

^a^Indicates patients with current infection by a specific influenza virus A subtype despite a normally protective titer of the relevant subtype-specific IgG antibodies

Of the 92 patients with a PCR-confirmed acute influenza A(H3N2) infection and for whom the HI-confirmed subtype-specific IgG serostatus was available, 78 (84.8%) had an “A(H3N2) primary infection” and 14 (15.2%) an “A(H3N2) re-infection”. Of the 78 patients with an “A(H3N2) primary infection”, 62 (79.5%) had no influenza virus A IgG at all and 16 (20.5%) had influenza virus A IgG against the other subtype, A(H1N1)pdm09 (14 of 16 with protective titers). Of the 14 “A(H3N2) re-infections”, 6 (42.9%) patients had IgG against only influenza A(H3N2) and 8 (57.1%) against both A(H3N2) and A(H1N1)pdm09. One patient with “A(H3N2) re-infection” had a usually protective titer against influenza A(H3N2); this child had a history of asthma and of respiratory tract complications. Four of the “A(H3N2) re-infection” patients had protective IgG titers against the other subtype A(H1N1)pdm09.

Regarding the 48 patients with PCR-confirmed acute influenza A(H1N1)pdm09 infection for whom the HI-confirmed subtype-specific IgG serostatus was available, there were 44 (91.7%) with “A(H1N1)pdm09 primary infections” and 4 (8.3%) with “A(H1N1)pdm09 re-infections”. Of the 44 patients with “A(H1N1)pdm09 primary infections”, 25 (56.8%) showed no IgG against any influenza virus A and 19 (43.2%) had IgG against the other subtype A(H3N2) (12 of 19 with protective titers). Of the 4 “A(H1N1)pdm09 re-infections”, only 1 (25%) patient had IgG solely against A(H1N1)pdm09 and 3 (75%) against both A(H1N1)pdm09 and A(H3N2). In one of these 4 patients with “A(H1N1)pdm09 re-infection”, the A(H1N1)pdm09 IgG titer was 1:80 and, thus, considered to be normally protective; this patient suffered from an underlying chronic condition. Two “A(H1N1)pdm09 re-infection” patients had protective IgG titers against the other subtype A(H3N2).

#### Serological status (IgG) of influenza B patients on the type level (ELISA)

Regarding the 39 patients with PCR-confirmed influenza virus B infection and with ELISA-confirmed IgG status, 38 (97.4%) had an “influenza B primary infection” and only a single patient had an “influenza B re-infection” (2.6%) on the type level.

#### Serological status (IgG) of influenza B patients on the lineage level (HI assay)

Lineage-specific influenza virus B IgG serostatus could be determined by HI assays for 35 (89.7% of 39) patients with PCR-confirmed influenza B infection. There were 26 “B/Yamagata primary infections”, one “B/Yamagata re-infection”, 8 “B/Victoria primary infections” and no “B/Victoria re-infections”. All 34 patients with lineage-specific primary infection showed no influenza virus B IgG against any of the two influenza B lineages. The single observed “B/Yamagata re-infection” occurred in a 4-year-old child, co-infected with both RSV and coronavirus, and with IgG against B/Yamagata at a normally protective level.

Due to the single influenza B patient with re-infection confirmed by ELISA/HI influenza B IgG serostatus, statistical comparisons of patients with influenza B primary infections and re-infections were not possible and, hence, all further analyses refer solely to the subsample of influenza A patients for whom ELISA/HI-confirmed influenza A IgG serostatus was available.

### Comparisons of sociodemographic and clinical characteristics of patients with influenza A primary infections or re-infections on the type and on the subtype level

#### Comparisons of type level “influenza A primary infections” versus “influenza A re-infections” (type-specific IgG serostatus ELISA-confirmed)

On the type level, comparison of the 87 “influenza A primary infections” and the 91 “influenza A re-infections” (Table 2) showed that children with “influenza A re-infection” were slightly older (median age 3.9 years) than children with “influenza A primary infection” (3.4 years; p = 0.016). Furthermore, they had a viral co-infection less often (22% of “influenza A re-infection” versus 36% of “influenza A primary infection”; p = 0.044). In univariate analyses, the severity of disease measured as median duration of fever plus cough/rhinitis (main outcome criterion) was 4 days (IQR 3–6) in children with “influenza A re-infection” compared to 3 days (IQR 3–5) in children with “influenza A primary infection” (p = 0.029). Multivariable analyses indicated a 19% increase (corresponding to a difference of less than 1 day) in the duration of the main outcome criterion for children with “influenza A re-infection” (p = 0.03). Duration of the single symptoms fever and of cough, respectively, was also longer (by a median duration of about 1 day or less) in children with “influenza A re-infection” (p = 0.044 and p = 0.025, respectively). All other severity assessments, clinical characteristics and healthcare-system related outcomes were similar.

**Table 2:** Disease characteristics of children presenting with acute influenza A primary infections or acute influenza A re-infections (regardless of influenza A subtype), with primary infections and re-infections determined on the type level by serological influenza virus-A IgG status (ELISA)

Characteristics of disease	Acute influenza A primary infections N = 87	Acute influenza A re-infections N = 91	p-value*
Socio-demographic/viral characteristics			
Age, in years (median, IQR)	3.4 (1.9–4.5)	3.9 (2.7–4.9)	**0.016**
Underlying chronic condition; n (%)	11 (12.6)	9 (9.9)	0.561
Influenza type/subtype distribution (current infection); n (%)			0.444
A(H3N2)	62 (71.3)	60 (65.9)	
A(H1N1)pdm09	25 (28.7)	31 (34.1)	
High influenza virus viral load (ct < 25); n (%)	42 (48.3)	42 (46.2)	0.777
Viral co-infection; n (%)	31 (35.6)	20 (22.0)	**0.044**
Duration of disease, maximum body temperature			
Days with fever + cough/rhinitis (MOM); median (IQR)	3 (3–5)	4 (3–6)	**0.029**
Days with fever; median (IQR)	4 (3–5)	4 (3–6)	**0.044**
Days with cough; median (IQR)	10 (6–12)	11 (8–13)	**0.025**
Days with rhinitis; median (IQR)	11 (8–14)	12 (9–15)	0.152
Maximum temperature; median (IQR)	39.8 (39.3–40.0)	39.7 (39.3–40.0)	0.777
Duration of disease; median (IQR)	8 (6–12)	9 (7–13)	0.119
Complications			
Occurrence of complications (acute otitis media or lower respiratory tract complication or febrile seizures); n (%)	25 (28.7)	20 (22.0)	0.300
CRP in mg/dl; median (IQR)	0.7 (0.2–1.5)	0.5 (0.2–1.6)	0.629
Severity assessment			
Physician assessment at practice visit as moderately/severely ill; n (%)	62 (73.8)	61 (70.9)	0.675
CARIFS Sum Score at day of practice visit (median, IQR)	30 (21–39)	30 (22–38)	0.703
CARIFS Sum Score at day 3 after practice visit (median, IQR)	19 (10–31)	19 (10–28)	0.670
CARIFS Sum Score at day 6 after practice visit (median, IQR)	7 (2–13)	9 (4–17)	0.334
Healthcare-system related outcomes			
Days in bed after practice visit (median, IQR)	0.5 (0.0–1.0)	0.0 (0.0–2.0)	0.829
Absenteeism from child care after practice visit, in days (median, IQR)	5 (3–6)	5 (3–7)	0.590
Parent workdays lost after practice visit (median, IQR)	4 (2–7)	3 (2–5)	0.453
Additional pediatric practice visit(s); n (%)	27 (31.0)	37 (40.7)	0.181
Additional specialist/emergency care/hospital visit; n (%)	4 (4.6)	6 (6.6)	0.563

Data from 178 PCR-confirmed influenza patients from pediatric practices in Bavaria (Germany), 2013–2015

*CARIFS* Canadian Acute Respiratory Illness and Flu Scale, *CRP* C-reactive protein, *ct* cycle threshold value, *IQR* Inter-quartile range, *MOM* main outcome measure

^*^Chi^2^ or Fisher’s Exact test, respectively, for categorical data; Mann–Whitney U-test for continuous data

For a subsample of 73 of the 87 children with “influenza A primary infection” and 83 of the 91 children with “influenza A re-infection”, a detailed history of previous diagnoses of the upper and lower respiratory tract was available. There were no significant differences between both groups regarding the frequency of previous chronic respiratory tract diagnoses (such as asthma, hypersensitivity of upper airways, or chronic obstructive lung disease; p = 0.915), or the proportion of children with at least one previous acute upper (p = 0.307) or lower respiratory tract diagnosis (p = 0.186). The average number of previous acute respiratory episodes per month (p = 0.877) and of the previous practice visits with a respiratory diagnosis were similar as well (p = 0.848).

#### Comparison of subtype level “A (H3N2) primary infections” versus “A(H3N2) re-infections” and “A (H1N1)pdm09 primary infections” versus “A(H1N1)pdm09 re-infections” (subtype-specific IgG serostatus HI-confirmed)

On the subtype level, univariate comparison of 78 (84.8% of 92) “A(H3N2) primary infections” vs. 14 (15.2% of 92) “A(H3N2) re-infections”, defined according to their HI-confirmed subtype-specific IgG serostatus, showed no significant differences in any investigated variable, including age (p = 0.832) and severity measured by duration of flu-like illness (main outcome criterion; “A(H3N2) re-infections” with a median of 4 days [IQR 3–5] vs. “A(H3N2) primary infections” with a median of 3 days [IQR 2–5]; p = 0.626) (Table 3).

**Table 3:** Disease characteristics of children presenting with acute “influenza A(H3N2) primary infections” or acute “influenza A(H3N2) re-infections”, with primary infections/re-infections determined on the subtype level, by serological influenza virus A(H3N2) IgG status (hemagglutination inhibition assay)

Characteristics of disease	Acute influenza A(H3N2) primary infections N = 78	Acute influenza A(H3N2) re-infections N = 14	p-value*
Socio-demographic/viral characteristics			
Age, in years (median, IQR)	3.8 (2.2–4.8)	3.4 (2.8–4.6)	0.832
Underlying chronic condition; n (%)	13 (16.7)	0 (0)	0.206
High influenza virus viral load (ct < 25); n (%)	39 (50.0)	3 (21.4)	0.078
Viral co-infection; n (%)	23 (29.5)	1 (7.1)	0.104
Duration of disease, maximum body temperature			
Days with fever + cough/rhinitis (MOM); median (IQR)	3 (2–5)	4 (3–5)	0.626
Days with fever; median (IQR)	3 (4–5)	4 (3–5)	0.899
Days with cough; median (IQR)	10 (6–13)	11 (7–14)	0.185
Days with rhinitis; median (IQR)	12 (9–14)	10 (7–13)	0.896
Maximum temperature; median (IQR)	39.7 (39.0–40.0)	39.8 (39.0–40.0)	0.959
Duration of disease; median (IQR)	9 (6–12)	9 (5–13)	0.736
Complications			
Occurrence of complications (AOM or lower respiratory tract complication or febrile seizures); n (%)	18 (23.1)	4 (28.6)	0.736
CRP in mg/dl; median (IQR)	0.7 (0.3–1.7)	0.7 (0.3–1.8)	0.707
Severity assessment			
Physician assessment at practice visit as moderately/severely ill; n (%)	56 (73.7)	7 (50.0)	0.111
CARIFS Sum Score at day of practice visit (median, IQR)	28 (19–38)	27 (23–32)	0.744
CARIFS Sum Score at day 3 after practice visit (median, IQR)	18 (10–30)	16 (4–22)	0.156
CARIFS Sum Score at day 6 after practice visit (median, IQR)	8 (3–12)	6 (0–14)	0.609
Healthcare-system related outcomes			
Days in bed after practice visit (median, IQR)	0 (0–1)	0 (0–2)	0.928
Absenteeism from child care after practice visit, in days (median, IQR)	5 (3–6)	4 (3–6)	0.684
Parent workdays lost after practice visit (median, IQR)	4 (2–5)	3 (3–4)	0.155
Additional pediatric practice visit(s); n (%)	23 (29.5)	3 (21.4)	0.750
Additional specialist/emergency care/hospital visit; n (%)	3 (3.8)	1 (7.1)	0.480

Data from 92 PCR-confirmed influenza patients from pediatric practices in Bavaria (Germany), 2013–2015

*CARIFS* Canadian Acute Respiratory Illness and Flu Scale, *CRP* C-reactive protein, *ct* cycle threshold value, *IQR* Inter-quartile range, *MOM* main outcome measure

^*^Chi^2^ or Fisher’s Exact test, respectively, for categorical data; Mann–Whitney U-test for continuous data

Comparison for 44 (91.7% of 48) “A(H1N1)pdm09 primary infections” vs. 4 (8.3% of 48) A(H1N1)pdm09 re-infections were limited by the low number of those re-infections (see Additional file 2: Table S1).

Due to the low numbers of re-infections for both subtypes, patients with subtype-specific primary infections/re-infections of both influenza A subtypes were pooled to compare the history of previous respiratory diagnoses. A subsample of 121 children were selected from the total 140 cases with PCR-confirmed influenza A subtypes and IgG seroprevalence status confirmed by HI-assay, based on the availability of complete medical information necessary for the subsequent analysis. In this subsample, there were 106 children with subtype-specific primary infections and 15 with subtype-specific re-infections. Of those children selected, 13% with subtype-specific primary infections and 33% with subtype-specific re-infections showed a previous diagnosis of asthma, hypersensitivity of upper airways or chronic obstructive lung disease (p = 0.060, not significant); all other comparisons did not indicate a trend.

## Discussion

Our study included 217 children 1 to 5 years of age presenting to pediatric practices with febrile, acute PCR-confirmed influenza, for whom ELISA-confirmed type-specific influenza IgG serostatus from serum samples collected at the day of the practice visit was available to determine previous influenza infections. The majority of these children were acutely infected with influenza virus A. Influenza B infections in our study sample were rare, in accordance with previous seroprevalence surveys from pediatric populations in Germany [18]. The proportion of PCR-confirmed influenza virus subtypes, with 56%/26%/18% for influenza A(H3N2)/A(H1N1)pdm09/B, reflected their circulation in the overall German population during the three observed seasons (average proportions 51%/26%/22% [30]). Independently from the respective influenza virus subtype detected, most children showed uncomplicated upper respiratory tract infections [8].

In the investigated age group, “influenza A primary infections” and “influenza A re-infections” with influenza A IgG serostatus confirmed by ELISA (defined regardless of the influenza A subtype involved) were observed in the pediatric practices with similar frequency. In contrast, almost all observed influenza B infections with ELISA-confirmed influenza B IgG serostatus were primary infections (97%). This was in accordance with previous seroprevalence studies from Germany, indicating that the first contact with influenza B usually occurs later in childhood than with influenza A [18]. Due to the low number of “influenza B re-infections”, comparative analyses of primary infections and re-infections had to be restricted to patients with influenza A.

Clinical severity of pediatric practice patients with “influenza A primary infections” and “influenza A re-infections” were similar, indicating that in the toddler and pre-school age group priming by natural influenza A infection per se might not necessarily mitigate severity of subsequent “influenza A re-infections”. Interestingly, children with “influenza A re-infections” even showed a slightly longer duration of fever plus cough/rhinitis (by about 1 day) than children with “influenza A primary infections”, as well as a slightly longer duration of the singular symptoms (cough, fever, rhinitis). This prolonged symptom duration in “influenza A re-infections” could not be explained by a higher “vulnerability in patients” with these re-infections due to underlying chronic conditions or their history of respiratory diagnoses.

In a second approach, we defined influenza A primary infections and re-infections based on the PCR-confirmed influenza subtype and using hemagglutination inhibition (HI) assays to confirm subtype-specific IgG status. The majority of the “influenza A re-infections” defined previously on the type level turned out to actually be primary infections with respect to a specific influenza A subtype (primary subtype infections), whereas subtype-specific influenza A re-infections were rare in this age group and accounted for only 13%. Hence, if parents of such young children seek outpatient treatment due to febrile influenza, their children would be usually experiencing their first-ever infection with a specific influenza A subtype, but not necessarily their first-ever infection with an influenza A virus. In a relevant proportion of these children, their IgG antibodies indicated that they already previously had (at least) a primary infection with the other influenza A subtype earlier in their lives.

In the 140 influenza A patients with HI-confirmed IgG serostatus analysed on the subtype level, the low number of subtype-specific re-infections observed in the pediatric practices suggests that subtype-specific re-infections either occurred only rarely in this age group or that the symptoms of most subtype-specific re-infections were too mild (oligo- or asymptomatic) for presentation at a pediatric practice. Nevertheless, for those children needing a pediatric practice visit the comparison between primary infections and re-infections on the subtype level—although limited by the low number of subtype-specific re-infections—suggests similar severity. Children with subtype-specific influenza A re-infections presenting in practices with febrile acute respiratory infection might belong to a specifically vulnerable group. Although they did not significantly differ from children with subtype-specific primary infections concerning the presence of underlying chronic conditions overall, they tended to have a history of respiratory diagnoses such as asthma, chronic obstructive lung disease, or hypersensitivity of upper airways more often.

Regarding the subtype-specific IgG serostatus based on HI-assay, repeated infections with influenza virus seem to occur frequently in pre-school children. From all 122 children with a subtype-specific influenza A primary infection, 29% experienced now their (at least) second infection with an influenza A virus. From 18 children with a subtype-specific influenza A re-infection, 61% already showed IgG against both influenza A subtypes, indicating that their acute infection was (at least) their third infection with influenza A. In addition, for 54 influenza A patients with HI-confirmed subtype-specific influenza virus A IgG titers, additional analyses revealed that 40 of these patients also had at least one lineage-specific IgG titer for influenza virus B. Four children (all aged 3–4 years) even possessed IgG not only against both influenza A subtypes but also against both influenza B lineages, indicating that they had currently experienced their (at least) fifth influenza virus-infection. A recent study from Austria also confirmed the occurrence of multiple (up to four) symptomatic influenza virus infections in otherwise healthy, unvaccinated children within four consecutive influenza virus seasons, but they did not determine the influenza virus subtype/lineage and assumed that these recurrent influenza virus infections were largely due to the different subtypes/lineages [31]. Nevertheless, due to the epidemiological situation in Austria, they suspected that some children in their study most likely had repeated influenza B infections, indicating no or limited lineage-specific cross-protection; in single children, they suspected even recurrent influenza B of the same lineage, suggesting only short-lasting immunity for this lineage. Our seroprevalence results from pre-school outpatient children now demonstrated that re-infections with the same influenza virus subtype requiring medical attention might indeed occur in some patients within a few influenza seasons. Such subtype-specific influenza A re-infections usually occur when the IgG titers elicited from previous infection(s) against the infecting subtype were too low for protection. This indicates that previous subtype-specific infection(s) in these children either were not able to induce a sufficiently high immune response to prevent these re-infections, or that the IgG antibodies derived from the previous infection(s) were waning rapidly. The presence of subtype-specific IgG with non-protective levels might even have contributed to the prolonged symptom duration of re-infections. As shown for other respiratory viruses, insufficient IgG antibodies for full protection in previously infected patients may result in a less effective immune response than in immunologically naïve patients [32]. In those rare cases in our study where children were re-infected by the same influenza A subtype despite a normally protective IgG titer against this same subtype, there were indications for higher “vulnerability”.

Subtype-specific influenza A primary infections and re-infections occurred even when the children had usually protective titers against the other influenza A subtype, which was the case in 23%. Hence, in these patients there was no indication of cross-protection among the two currently circulating influenza A subtypes. Influenza A(H1N1)pdm09 and A(H3N2) belong to different antigenic groups, and cross-protection among subtypes is expected to occur mainly within each antigenic group [20]. This is also in accordance with previous immunological studies claiming a narrow immunological response to influenza virus infections in children [14].

For influenza A(H1N1)pdm09, it has been assumed that older adults are largely protected against this pandemic virus by pre-existing cross-reactive antibodies due to a 1918 pandemic-like A(H1N1) infection early in their lives [17, 20]. Such an age-dependent impact shaping the epidemiological pattern of current seasonal influenza epidemics may only be observable as of now for the present older population. For them, there was a clear distinction in the exposure to the subtypes: in the time following 1918, only influenza A(H1N1) circulated, followed by the emergence of A(H3N2) in 1968. Since 1977, however, A(H1N1) and A(H3N2) co-circulate, so children are now exposed to both subtypes. Modeling studies suggested that this early-life exposure might result in better protection to both these subtypes when these age cohorts reach old age [20].

Presently, parents and pediatricians should be aware of the fact that recurrent, clinically relevant influenza infections do occur frequently in pre-school children and that confirmed influenza illness in such young children does not rule out a similarly severe influenza infection during the subsequent influenza seasons.

### Strengths and limitations

To our knowledge, this study is the first comparing clinical characteristics of natural influenza virus primary infections and re-infections in children. The strengths of our study were the prospective and multi-center design, active screening of patients with acute respiratory infections for influenza virus, PCR-confirmation of influenza virus infections, and determination of influenza virus IgG serological status even to the subtype level.

Our limitations were the very low number of influenza B re-infections and subtype-specific influenza A re-infections found in the investigated age group. Regarding protective titers on the subtype level, the influenza virus strains used in the hemagglutination inhibition (HI) assay may not have covered other strains of the same subtype in some patients. Five of the 14 “influenza A(H3N2) re-infections” were observed during the influenza season 2014/2015 and were likely infected with an A(H3N2) subtype variant emerging that season; hence, they could be considered “primary infections” on the subtype variant level. Due to the cross-sectional design, severity of influenza virus primary infections and re-infections were only comparable among groups of patients but did not allow a direct comparison per patient. Thus, longitudinal, prospective cohort studies on healthy children followed from birth onwards are needed to evaluate the individual history of influenza infections and immunity. Finally, our study was focused on medically relevant influenza virus infections in outpatient locations, defined as febrile acute respiratory illness. To discover the full spectrum of clinical presentations regarding influenza virus primary infections and re-infections, further studies on this topic should include children with non-febrile acute respiratory infections treated in pediatric practices as well as children with acute respiratory infections who do not require a pediatric practice visit at all. A particularly interesting question is whether influenza virus-associated hospitalizations in otherwise healthy children may result from subtype-specific influenza virus primary infections.

## Conclusions

Pre-school children with febrile acute respiratory infection due to “influenza A primary infections” and “influenza A re-infections” presented with similar frequency in pediatric practices. Taking subtype-specific influenza virus A IgG antibody serotyping into account, influenza infections requiring medical attention in this age group were almost all primary infections for a specific influenza A subtype. Children with subtype-specific re-infections needing medical attention were rare at this age and might belong to a specifically vulnerable group whereas the majority of children might develop symptoms too mild for a pediatric practice visit due to partial immunity.

Unexpectedly, comparisons of “influenza A primary infections” and “influenza A re-infections” defined on the type level as well as on the subtype level indicated a similar or even slightly longer duration of fever and/or respiratory symptoms for re-infections. Children with subtype-specific re-infections usually had non-protective IgG titers against the infecting subtype, suggesting only short-time humoral immunity induced by the previous infection with this subtype. Children developed infections with a specific influenza A subtype despite having protective titers of IgG antibodies against the other influenza A subtype. Thus, at least in these young children requiring medical attention for influenza virus disease, there was no or only limited heterosubtypic cross-protection.

The significance of these findings in medically treated outpatients and the overall burden of influenza primary infections and re-infections in children should be investigated in population-based studies that also include children with asymptomatic and oligo-symptomatic influenza virus infection, or in long-term longitudinal studies starting from birth. Overall, the role of natural primary infections in an immunologically naïve population and its impact on short-term and long-term immunity, as well as the severity of natural primary infections and re-infections in different age groups deserve more attention.

## Supplementary Information

**Additional file 1: Supplementary Methods.** Information on primers and probes used for PCR.

**Additional file 2: Supplementary Table S1.** Disease characteristics of children presenting with acute influenza “A(H1N1)pdm09 primary infections” or with acute “A/H1N1)pdm09 re-infections”.

## Data Availability

The datasets generated and analysed in this study are not publicly available. This is due to data protection regulations, contracts for participating practices and informed consent form for parents, which guaranteed to all participants that individual patient data will be collected only for the purpose of the study, will be analysed solely at the Department of Pediatrics, University Hospital of Würzburg, and will not be forwarded to any third party. Fully anonymized data will be available from the corresponding author upon reasonable request, as far as permitted by the Office for Data Protection and the Legal Department of the University Hospital Würzburg.
