# Supplementary material for: Similar severity of influenza primary and re-infections in pre-school children requiring outpatient treatment due to febrile acute respiratory illness: prospective, multicentre surveillance study (2013–2015)
Source: BMC Infect Dis. 2022 Jan 4;22:12. doi: 10.1186/s12879-021-06988-7 (PMC8724639; doi:10.1186/s12879-021-06988-7)
Supplement: Supplementary file 1 — Additional file 1: Supplementary Methods. Information on primers and probes used for PCR. [file 12879_2021_6988_MOESM1_ESM.docx]

**Additional File**

**Methods**

Additional information:

Primers and probes used by the commercial kit “FTD® Respiratory Pathogens 21’, Fast Track Diagnostics” were not disclosed by the company. For H3-specific PCR, primers and probes INA_H3h-1070FW, INA_H3h-1144dProbe, INA_H3h-1167Rv were used according to recommendations by the WHO:

<https://www.who.int/influenza/gisrs_laboratory/WHO_information_for_the_molecular_detection_of_influenza_viruses_20171023_Final.pdf>
