# Supplementary material for: Similar severity of influenza primary and re-infections in pre-school children requiring outpatient treatment due to febrile acute respiratory illness: prospective, multicentre surveillance study (2013–2015)
Source: BMC Infect Dis. 2022 Jan 4;22:12. doi: 10.1186/s12879-021-06988-7 (PMC8724639; doi:10.1186/s12879-021-06988-7)
Supplement: Supplementary file 2 — Additional file 2: Supplementary Table S1. Disease characteristics of children presenting with acute influenza “A(H1N1)pdm09 primary infections” or with acute “A/H1N1)pdm09 re-infections”. [file 12879_2021_6988_MOESM2_ESM.docx]

**Additional File**

**Table S1: Disease characteristics of children presenting at a pediatric practice with acute influenza „A(H1N1)pdm09 primary infections“ or acute „A(H1N1)pdm09 re-infections“, with primary infections/re-infections determined by the serological IV-A(H1N1)pdm09 IgG status.**

| **Characteristics of disease** | **Acute influenza A(H1N1)pdm09**  **primary infections**  **N=44** | **Acute influenza A(H1N1)pdm09**  **re-infections**  **N=4** |
| --- | --- | --- |
| **Socio-demographic / viral characteristics** |  |  |
| Age, in years (median, IQR) | 3.4 (2.0-5.0) | 3.9 (3.4-5.4) |
| Underlying chronic condition; n (%) | 0 (0) | 1 (25.0) |
| High Influenza virus viral load (ct <25); n (%) | 21 (47.7) | 3 (75.0) |
| Viral co-infection; n (%) | 19 (43.2) | 0 (0.0) |
| **Duration of disease, maximum body temperature**** |  |  |
| Days with fever +cough/rhinitis (MOM); median (IQR) | 4 (3-6) | 7 (4-10) |
| Days with fever; median (IQR) | 4 (3-6) | 7 (4-10) |
| Days with cough; median (IQR) | 10 (8-13) | 13 (11-15) |
| Days with rhinitis; median (IQR) | 12 (7-15) | 15 (15-17) |
| Maximum temperature; median (IQR) | 39.8 (39.4-40.0) | 39.6 (39.3-40.4) |
| Duration of disease; median (IQR) | 8 (6-12) | 11 (4-15) |
| **Complications** |  |  |
| Occurrence of complications (Acute otitis media or lower respiratory tract complication or febrile seizures); n (%) | 12 (27.3) | 0 (0.0) |
| CRP in mg/dl; median (IQR) | 0.5 (0.1-1.3) | 0.3 (0.3) |
| **Severity assessment** |  |  |
| Physician assessment at practice visit as moderately / severely ill; n (%) | 30 (71.4) | 3 (100.0) |
| CARIFS Sum Score at day of practice visit (median, IQR)*** | 32 (23-38) | 38 (32-43) |
| CARIFS Sum Score at day 3 after practice visit (median, IQR)*** | 18 (7-28) | 27 (13-41) |
| CARIFS Sum Score at day 6 after practice visit (median, IQR)*** | 7 (3-13) | 19 (12-26) |
| **Healthcare-system related outcomes** |  |  |
| Days in bed after practice visit (median, IQR) | 0 (0-2) | 1 (0-2) |
| Absenteeism from child care after practice visit, in days (median, IQR) | 5 (3-6) | 9 (4-9) |
| Parent workdays lost after practice visit (median, IQR) | 4 (3-5) | 1 (1-1) |
| Additional pediatric practice visit(s); n (%) | 15 (34.1) | 1 (25.0) |
| Additional specialist / emergency care / hospital visit; n (%) | 3 (6.8) | 0 (0.0) |

Data from 48 PCR-confirmed influenza patients from pediatric practices in Bavaria (Germany), 2013-2015.No statistical comparison due to low number of patients with „A(H1N1) re-infections“. CARIFS = Canadian Acute Respiratory Illness and Flu Scale; CRP = C-reactive protein; ct =cycle threshold value IQR: Inter-quartile range; MOM = main outcome measure,
